# Four Cases of Fibroma

**Published:** 1880-07

**Authors:** N. F. Thompson


### IV.—NOTES OF FOUR CASES OF FIBROMA.

COLLECTED FROM THE CLINICS OF
THOS. H. HAWKINS, M. D. V. S.,
PROFESSOR OF SURGERY IN COLUMBIA VETERINARY COLLEGE, ETC.

BY N. F. THOMPSON, D. V. S.

Unfortunately, in veterinary practice, great difficulty is found in obtaining a good clinical history, but such as was obtainable is given below.

CASE I.—A sorrel gelding, nine years old, fifteen and a half hands high, of a nervo-lymphatic temperament, was brought to the hospital for surgical treatment. On inspection there was found in the right pectoral region, about eight inches above, and to the right of the ensiform cartilage of the sternum, and six inches above the scapulo-humeral articulation, a large cup-shaped swelling, longitudinal diameter seven and a half inches, transverse diameter five and three-quarter inches. On manipulation it was found to be firm to the touch, immovable, and adherent to the muscular tissue, of which apparently it formed a part, but not attached to the skin.

ANATOMICAL SITUATION.—The muscles chiefly involved were the mastoido-humeralis, which served to give origin to the greater part of the tumor, the sterno-prescapularis and outer margin of the anterior spinatus muscle. The fibroid growth dipped down into the interstices between the mastoido-humeralis and sterno-trochineus muscles.

THE CLINICAL HISTORY.—Tumor was first noticed twelve weeks prior to animal's reception in the hospital. The animal had been working before a milk wagon, and with an ill-fitting collar. One night a small abrasion was noticed, and a puffy condition of the tissues. The abraded surface was washed with salt and water, and the animal was left for the night. In the morning the entire pectoral region was the seat of œdematous swelling, and was extremely painful to the touch. The swelling gradually abated during the week but left over the seat of the present growth a doughy swelling, which finally changed to one of a firmer consistency, and steadily increased in size until a week prior to admission in the hospital, when the growth seemed checked. Diagnosis, fibroid tumor. Treatment, excision.

OPERATION.—Animal was placed in the stocks, and the usual means of restraint employed. An elliptical incision was made both sides of the median line through the skin. The flaps were then dissected back to the base of the tumor, and held by means of sutures passed through the skin; the tumor was then dissected out. It was found to be adherent to the trachea and œsophagus, and the right jugular vein passed over the apex of the tumor, to which it was firmly bound by connective tissue. The vein was dissected away from the tumor and pressed to one side. Owing to the close proximity of important vessels, great care was required during the entire operation. It was found necessary to ligate a large branch of the left carotid artery, owing to its intimate connection with the substance of the tumor, as well as several small arteries which passed into the growth. The bands of connective tissue binding the tumor to the trachea and œsophagus were broken down by the handle of a scalpel, and the tumor removed entire. The cavity was then thoroughly cleansed with carbolic lotion, and the muscular tissue brought together by means of deep-seated sutures, seven in number. The skin was approximated by means of interrupted sutures, five in number, after which the parts were covered by cloths saturated in carbolic acid solution, and laxative food was ordered. Time of operation, one hour and forty-five minutes.*

RESULTS.—Rigors supervened ten hours after operation, followed by an increased temperature; pulse rose to 60, full and strong; respiration increased in frequency, and labored; 24 to the minute. Temperature, 104°. Symptoms were subdued with stimulants, followed by febrifuge drenches. Twenty-four hours after, pulse slightly increased in frequency; respiration norma'; temperature, 101 3-5; lips of the wound slightly thickened, œdematous; animal looking and feeling well; appetite good. Forty-eight hours after, entire pectoral region was the seat of general œdematous swelling, but no constitutional disturbance. Seventy-two hours after, swelling had increased in volume, and was more diffuse, dropping down into the axillary reigion; lips of wound thickened and everted, sutures giving way, and a s ight discharge of creamy looking pus. Treatment, carbolic lotion applied to the part. Ninety-six hours after, swelling further increased, extending down to the knee; superficial sutures ruptured, lips of wound gaping, profuse discharge of a yel owish pus, slightly fœtid in odor. Treatment continued. Fifth day, discharge very profuse of a yellowish green co'or and very fœtid, swelling in the arm and axillary regions gradually abating, lips of the wound wide'y g ping and granulating. Treatment, 2 per cent. solution of chloride of zinc to be used as a spray twice a day. This treatment was continued until the twentieth day, during which time the swelling gradually abated, and the cavity filled up with granular material, when the animal was discharged, with parts a'most restored to their normal condition. Recovery complete.

---

CASE II.—B ack mare, ten years old, fourteen and a half hands high.

HISTORY.—Four weeks previously owner noticed a small nodular growth, which had gradually increased in size up to the present time. There had never been tenderness or heat in the part, and the animal had suffered no inconvenience. The growth was situated in the inferior cervical region, a little to the left of the median line over the region of the transverse process of the fifth cervical vertebra, and was deeply imbedded in the mastoido-humeralis muscle, and seemed very much like hypertrophy. It was firm to the touch, and adherent to the muscular tissue, but not to the cuticle. Longitudinal diameter, 4½ inches; transverse, 6 inches.

TREATMENT.—Attempts were at first made to discuss the growth by counter irritation and a seton, which proving ineffectual, it was decided to extirpate.

OPERATION.—The animal being cast and properly secured, an incision was made through the integument, which was then dissected back and the tumor sliced off

*Weight of tumor 5½ pounds. Microscopical report not rendered.

from its base, leaving only soft normal tissue beneath. The lips of the wound were then drawn together and retained in position by means of quill sutures. Carbolized dressings were then applied. The wound healed kindly, with very little discharge. Time of operation, 45 minutes; weight of tumor, 2 lbs.; character, fibroma. Animal discharged in five weeks, fit for work.

---

CASE III.—Buckskin gelding, ten years old, sixteen hands high.

HISTORY.—First noticed tumor three weeks prior to admission in the hospital; growth was steady from that time. Tumor painful on pressure, oval in shape, soft and movable. Locality, about four inches above the scapulo-humeral articulation, on the right side and over the seat of the mastoido-humeralis and anterior spinatus muscles.

OPERATION.—Incision was made in the median line, cellular tissue was then broken down with the handle of the scalpel, and the tumor enucleated. It was found to be lodged in the cellular tissue principally, and but slightly attached to the ante spinatus muscle. The parts were cleansed with carbolized dressing, the lips of the wound were brought together with the quill suture, and carbolized dressings applied. Wound healed kindly, and the animal pronounced fit for work in two weeks. Time of operation, thirty minutes; character of the tumor, fatty fibroma; weight was 1½ pounds.

---

CASE IV.—Sorrel gelding, seven years old, fifteen-three hands high, of a nervous temperament.

HISTORY.—The animal was castrated two years previous. This operation was performed with the animal in a standing position; wound healed kindly. Seven months afterwards a thickening was noticed on the left side of the scrotum in the inferior portion, about one inch above the median raphæ. This thickening gradually became greater, and on manipulation, the end of the spermatic cord could be felt indurated and adherent. A peculiar feature in this history was, that after exercise, the entire scrotum would swell as would also the *left hind leg* from the hip to the hock. The swelling was soft, would pit on pressure, and would last from ten to twenty-four hours, when it would disappear. Three months previous to admission, an incision was made through the scrotum, and that part of the spermatic cord which was adherent thereto was dissected off.

DIAGNOSIS.—Fatty fibroma in the testicular envelopes. Treatment, excision.

OPERATION.—Animal was cast and secured properly, after which he was placed on his back with hind legs drawn forward. An elliptical incision was then made over the seat of the growth and the tumor dissected out. The edges of the wound were then brought together by means of interrupted sutures, and the parts were dressed with strong solution of carbolic acid in order to check hemorrhage, and also for its antiseptic effect. The sheath placed in a sling, and animal ordered to be kept quiet with light diet. Animal was removed two days after operation.

Character of tumor: Fatty fibroma, weight twenty ounces; time of operation, forty minutes.

